# Age-related Changes in Eye, Brain and Visuomotor Behavior in the DBA/2J Mouse Model of Chronic Glaucoma

**DOI:** 10.1038/s41598-018-22850-4

**Published:** 2018-03-15

**Authors:** Xiao-Ling Yang, Yolandi van der Merwe, Jeffrey Sims, Carlos Parra, Leon C. Ho, Joel S. Schuman, Gadi Wollstein, Kira L. Lathrop, Kevin C. Chan

**Affiliations:** 1 NeuroImaging Laboratory, University of Pittsburgh, Pittsburgh, Pennsylvania United States; 2 UPMC Eye Center, Eye and Ear Institute, Ophthalmology and Visual Science Research Center, Department of Ophthalmology, University of Pittsburgh, Pittsburgh, Pennsylvania United States; 3 Department of Bioengineering, Swanson School of Engineering, University of Pittsburgh, Pittsburgh, Pennsylvania United States; 4 Department of Electrical and Electronic Engineering, The University of Hong Kong, Pokfulam, Hong Kong China; 5 NYU Langone Eye Center, Department of Ophthalmology, NYU School of Medicine, NYU Langone Health, New York University, New York, New York, United States; 6 Department of Radiology, NYU School of Medicine, NYU Langone Health, New York University, New York, New York, United States

## Abstract

Although elevated intraocular pressure (IOP) and age are major risk factors for glaucoma, their effects on glaucoma pathogenesis remain unclear. This study examined the onset and progression of glaucomatous changes to ocular anatomy and physiology, structural and physiological brain integrity, and visuomotor behavior in the DBA/2J mice via non-invasive tonometry, multi-parametric magnetic resonance imaging (MRI) and optokinetic assessments from 5 to 12 months of age. Using T2-weighted MRI, diffusion tensor MRI, and manganese-enhanced MRI, increasing IOP elevation at 9 and 12 months old coincided with anterior chamber deepening, altered fractional anisotropy and radial diffusivity of the optic nerve and optic tract, as well as reduced anterograde manganese transport along the visual pathway respectively in the DBA/2J mice. Vitreous body elongation and visuomotor function deterioration were observed until 9 months old, whereas axial diffusivity only decreased at 12 months old in diffusion tensor MRI. Under the same experimental settings, C57BL/6J mice only showed modest age-related changes. Taken together, these results indicate that the anterior and posterior visual pathways of the DBA/2J mice exhibit differential susceptibility to glaucomatous neurodegeneration observable by *in vivo* multi-modal examinations.

## Introduction

Glaucoma, the leading cause of irreversible blindness in the world^1,2^, is characterized by progressive degeneration of retinal ganglion cell projections to the brain^3,4^. Prevalence of this age-related disease is expected to increase in coming years due to aging populations, yet we still have no clear understanding of the underlying cause of the disease. Although age and intraocular pressure (IOP) elevation are the major risk factors for the disease^3^, their specific involvements in glaucoma pathogenesis in the visual system are unclear. In addition, recent studies suggest that glaucoma may continue to progress in some patients even after lowering IOP to population-derived normal levels, indicating that other key factors are contributing to the disease^5^. The clinically approved glaucoma treatment is only oriented at lowering IOP^6,7^, yet neuroprotective or neurorestorative interventions to glaucoma remain elusive, mainly due to the unclear mechanisms leading to the onset and progression of the disease^8,9^.

The DBA (“dilute brown non-agouti”) mouse line is a commonly used animal model for hereditary glaucoma^10^. Specifically, DBA/2J (D2) mice represent late onset, chronic pigmentary glaucoma due to elevations in IOP that result from melanosomal protein mutations. A tyrosinase-related protein 1 (Tyrp1) mutation causes iris stromal atrophy, while a premature stop codon in glycosylated protein nmb (Gpnmb) drives iris pigment dispersion^11^. Together, this results in pigment and cell debris blocking drainage of fluid within the eye and increasing IOP over time, and allows the D2 mouse to model many pertinent features of neurodegeneration in studying glaucoma’s pathogenesis and potential avenues for treatment^12,13^. Non-invasive evaluation of increasing IOP and the related pathogenesis in the eye and the brain’s visual pathway offers the potential for unraveling the mechanisms of glaucoma progression. In this study, we utilized tonometry, multi-parametric magnetic resonance imaging (MRI) and behavioral assessments to non-invasively probe the onset of glaucomatous changes and their progression by longitudinally measuring IOP, ocular anatomy, structural and physiological brain integrity, and visuomotor function in the D2 mouse model of chronic glaucoma.

*In vivo* MRI has been increasingly used at high magnetic field strengths to evaluate the rodent eye and brain without depth limitation^14–21^. Here, we tested whether anatomical T2-weighted MRI could detect changes in anterior chamber depth, vitreous body depth, and axial length in the eyes of D2 mice. Apart from ocular MRI, diffusion tensor MRI (DTI) non-invasively measures the anisotropy of water diffusion along the visual pathway^14,15^. For example, among the DTI-derived parameters, fractional anisotropy reflects the directionality and overall microstructural integrity of the brain, whereas axial and radial diffusivities have been reported to be sensitive to axonal and myelin integrity, respectively, in the white matter^14^. On the other hand, manganese (Mn)-enhanced MRI has been characterized as a robust technique for *in vivo* neuronal tract tracing and for examining the integrity of axoplasmic transport due to the paramagnetic nature of the Mn ions and their ability to undergo active anterograde axonal transport^15,18^. In this study, DTI and Mn-enhanced MRI were chosen to examine the structural organization and the physiological anterograde transport along the visual pathway of D2 mice, respectively, along with tonometry and behavioral tasks. Examinations were performed on D2 and age-matched wild type C57BL/6J (B6) mice. These *in vivo* imaging findings may help determine if glaucoma involves early pathophysiological events in both the eye and the brain, and whether such events progress with age and IOP elevation.

## Results

### IOP elevation progressed with age in D2 mice

**Figure 1:**
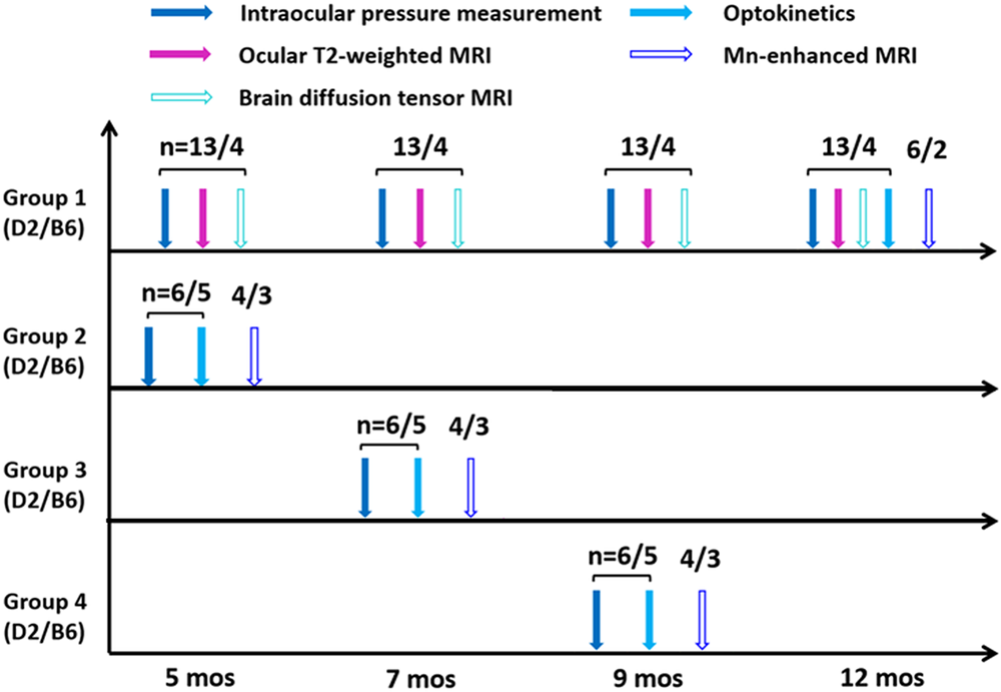
Experimental outline for measuring eye, brain and behavioral changes in the D2 mouse model of chronic glaucoma and the wild type B6 mice across age. Vertical arrows on the horizontal timelines represent intraocular pressure (IOP) measurements by tonometry (solid blue), ocular anatomical T2-weighted MRI (solid purple), brain diffusion tensor MRI (DTI) (hollow cyan), visuomotor behavioral assessments by optokinetics (solid light blue), and manganese (Mn)-enhanced MRI of anterograde transport (hollow dark blue). The numbers on top of the vertical arrows represent the sample size (n) of the D2 or B6 mice for each modality at each age. In Group 1, additional IOP measurements were performed in between 7, 9 and 12 mos for more precise longitudinal monitoring of the progression of IOP elevation in the same animals (Fig. 2a).

**Figure 2:**
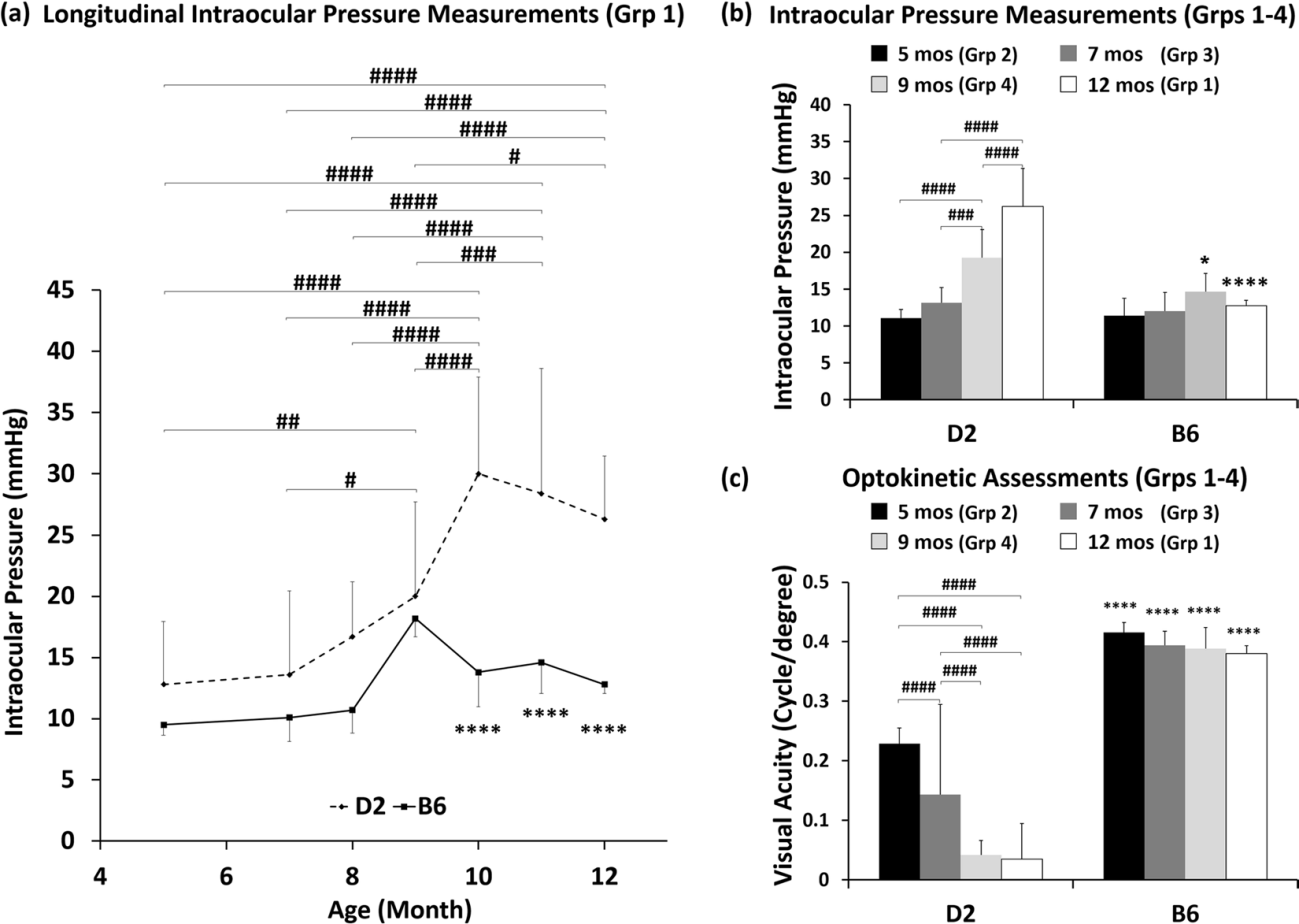
Intraocular pressure (IOP) measurements and optokinetic assessments of D2 and B6 mice across age. (**a**) IOP profiles of the D2 (dashed line) and B6 (solid line) mice in Group 1 from 5 to 12 mos. **(b**) IOP profiles in Groups 1–4 at 5, 7, 9 and 12 mos respectively. **(c)** Visual acuity of the D2 and B6 mice at 5 to 12 mos via optokinetic visuomotor behavioral assessments. Post-hoc Tukey’s tests between ages in the D2 mice: ^#^p < 0.05, ^##^p < 0.01, ^###^p < 0.001, ^####^p < 0.0001; between D2 and B6 mice: *p < 0.05; ****p < 0.0001. No significant difference was observed between ages in the B6 mice. Error bars represent ± standard deviation.

In this study, D2 and B6 mice were assigned to four groups as shown in Fig. 1. The groups were organized such that Group 1 underwent longitudinal non-invasive tonometry and imaging measurements before endpoint Mn injection at 12 months of age, while Groups 2–4 were only involved in endpoint experiments at 5, 7 and 9 months of age, respectively. Longitudinal measurements showed that the IOP in the D2 mice began to increase at about 8–9 months old (mos), reached its peak at 10 mos, and remained significantly higher than the baseline until the end of the experimental period at 12 mos (Fig. 2). The B6 mice in Group 1 had no significant IOP changes between time points, yet showed a trend of slight IOP increase in the older individuals, consistent with normal age-related changes reported in a previous study^22^. Groups 2–4 showed a similar pattern as Group 1 (Fig. 2b).

### Visuomotor function deteriorated in D2 mice during initial IOP elevation

Optokinetic behavioral assessments were performed by presenting mice with drifting sinusoidal gratings of varying spatial frequency, with the highest spatial frequency eliciting a behavioral response being identified as the animal’s visual acuity. In this study, a significant age-related decrease of visual acuity was observed in D2 mice assigned to Groups 1–4, averaging 0.23 cycle/deg at 5 mos, 0.14 cycle/deg at 7 mos, and a mere 0.04 cycle/deg at 9 mos (Fig. 2c). B6 mice, by contrast, outperformed the D2 mice and preserved a high visual acuity of about 0.4 cycle/deg regardless of their age.

### The anterior chamber elongated at a different rate than the vitreous body in D2 mice parallel to IOP increase and age

**Figure 3:**
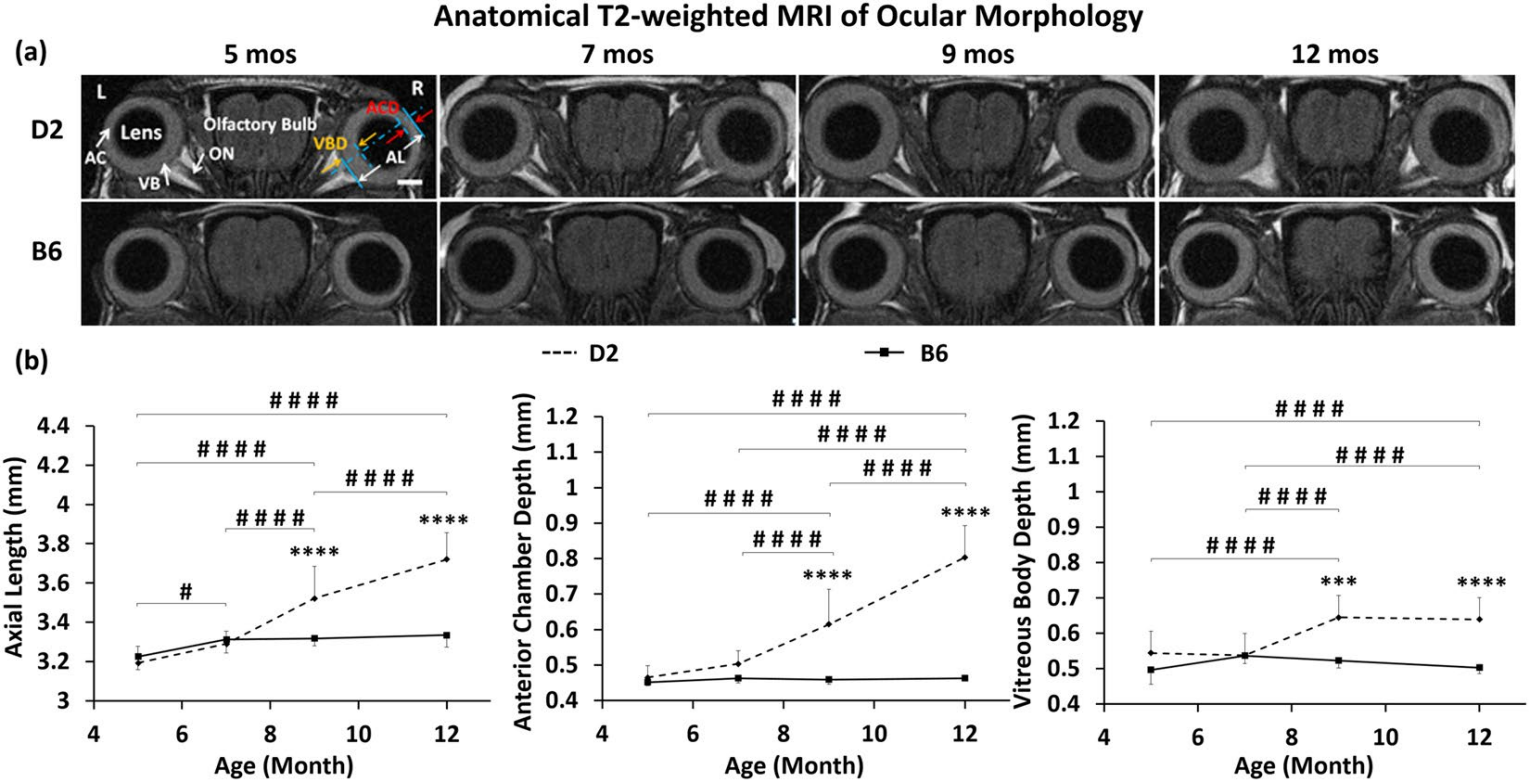
Anatomical T2-weighted MRI of ocular morphology in Group 1 across age. **(a**) Ocular anatomy, including the anterior chamber (AC), lens, vitreous body (VB) and optic nerve (ON), was visualized and qualitatively assessed in both eyes, and ocular dimensions including anterior chamber depth (ACD), vitreous body depth (VBD) and axial length (AL) were measured. Scale bar = 1 mm. (**b**) Quantitative comparisons of AL (left), ACD (middle) and VBD (right) in the D2 (dashed line) and B6 (solid line) mice across age. Post-hoc Tukey’s tests between ages in D2: ^#^p < 0.05, ^####^p < 0.0001; between D2 and B6 mice: *p < 0.05, **p < 0.01, ***p < 0.001, ****p < 0.0001. No significant difference was observed between ages in the B6 mice. Error bars represent ± standard deviation.

Ocular T2-weighted MRI showed a continuous increase in the ocular axial length of Group 1 D2 mice throughout the experimental period from 5 to 12 mos (Fig. 3). Examination of individual ocular compartments at 9 mos revealed an increase of anterior chamber depth and vitreous body depth along with IOP increase. Further elongation of the anterior chamber was observed at 12 mos along with further elevation of IOP. However, no significant change was observed in the vitreous body in D2 mice between 9 and 12 mos. No significant difference in axial length, anterior chamber depth or vitreous body depth was observed in the B6 mice across age.

### Optic nerve and optic tract integrity was compromised to different extents as IOP increased in D2 mice

**Figure 4:**
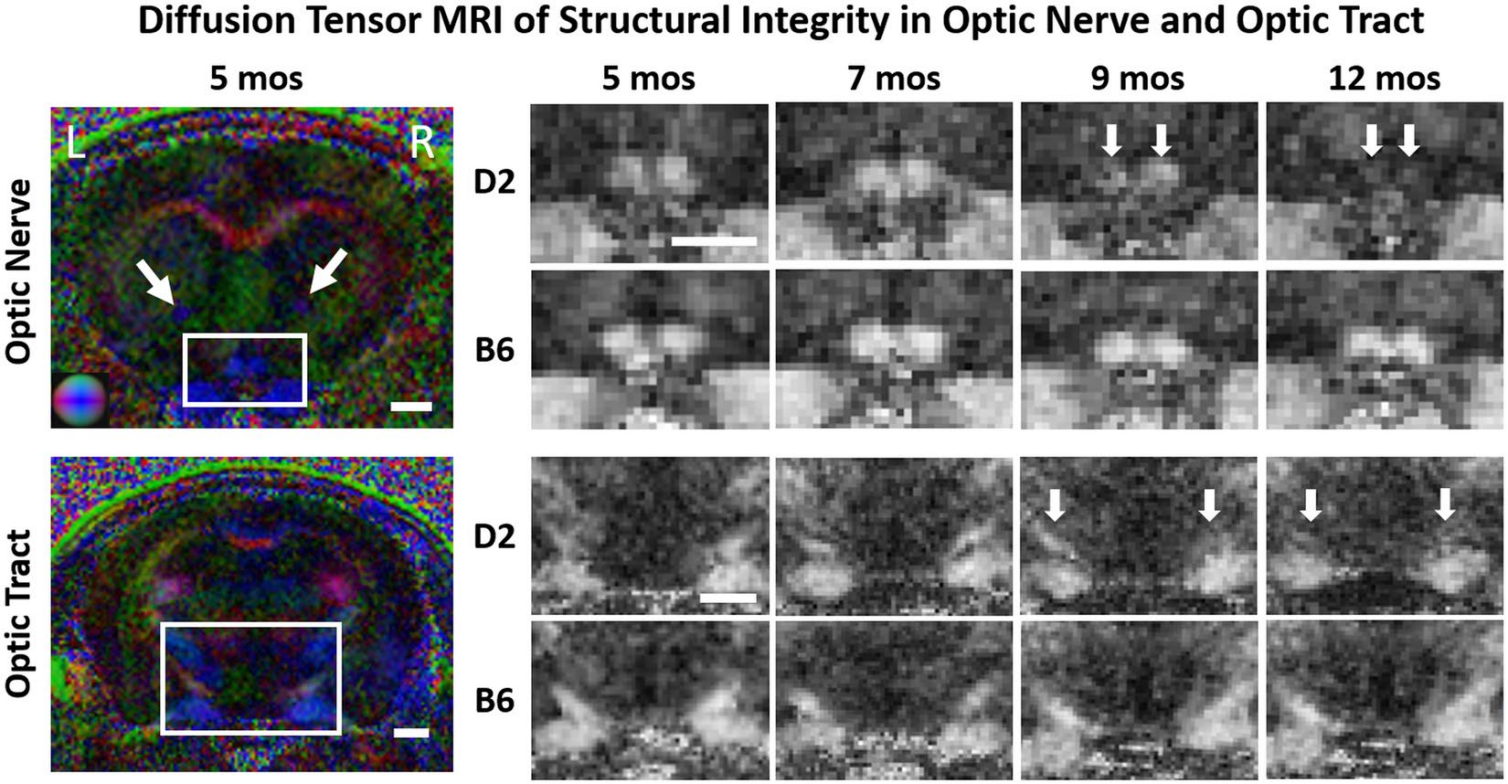
Diffusion tensor MRI (DTI) of structural integrity along the visual pathway of D2 and B6 mice in Group 1 across age. (Left) Whole-brain color-encoded fractional anisotropy (FA) directionality maps at the levels of the prechiasmatic optic nerve (top) and the optic tract (bottom) in a representative D2 mouse at 5 mos. White arrows indicate the anterior commissure. Color representations for the principal diffusion directions: blue, caudal-rostral; red, left-right; green, dorsal-ventral. (Right) FA value maps enlarged from the white boxes in the whole-brain color-encoded FA maps in the D2 and B6 mice across age. Note the different extents of FA decrease at 9 and 12 mos (arrows) relative to 5 and 7 mos in the D2 mice. Scale bar = 1 mm.

**Figure 5:**
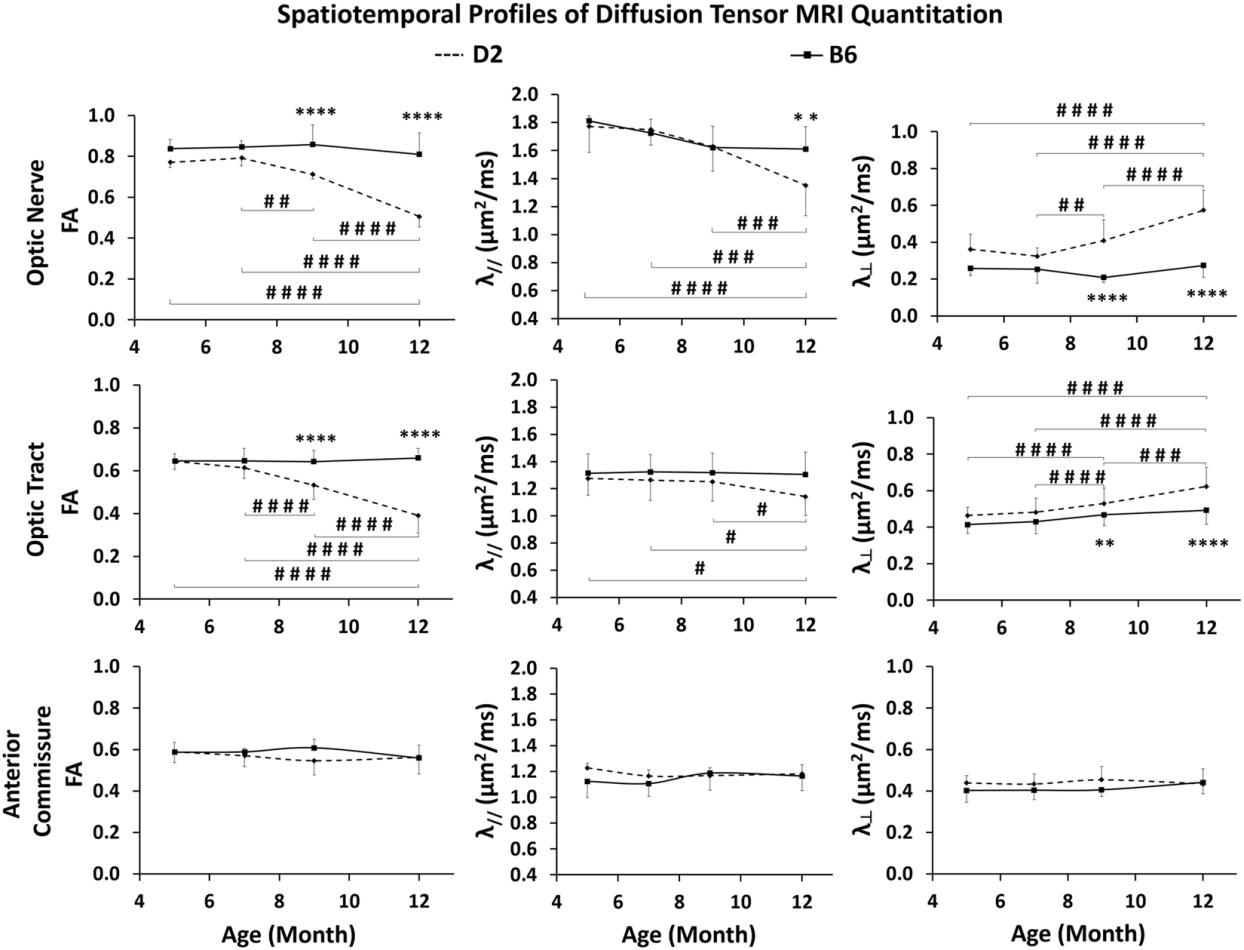
Quantitative spatiotemporal DTI profiles of the prechiasmatic optic nerve (top row), optic tract (middle row), and anterior commissure (bottom row) in the D2 and B6 mice. λ_//_: axial diffusivity; λ_⊥_: radial diffusivity. Post-hoc Tukey’s tests between ages in the D2 mice: ^#^p < 0.05, ^##^p < 0.01, ^###^p < 0.001, ^####^p < 0.0001; between D2 and B6 mice: *p < 0.05, **p < 0.01, ***p < 0.001, ****p < 0.0001.; No significant difference was observed between ages in the B6 mice. Error bars represent ± standard deviation.

Longitudinal DTI examination of the structural integrity of the brain’s visual pathway showed significant fractional anisotropy decrease and radial diffusivity increase starting at 9 mos (Figs 4 and 5), affecting both the optic nerve and optic tract of D2 mice. These changes progressed further along with the increasing IOP elevation at 12 mos. The corresponding axial diffusivity only showed a significant decrease at 12 mos. The directional diffusivities (i.e. radial and axial diffusivities) appeared to exhibit larger longitudinal changes in the optic nerve than the optic tract of the D2 mice. This effect was particularly marked at 12 mos, with axial diffusivity being changed by 23.78% and 10.5% in the optic nerve and optic tract respectively relative to 5 mos, and radial diffusivity being changed by 58.59% and 34.17% in the optic nerve and optic tract respectively relative to 5 mos (Fig. 5). No apparent DTI parametric change was observed in the anterior commissure of D2 mice across age (Fig. 5), and no apparent DTI parametric change was observed in the visual pathway or anterior commissure of B6 mice across age.

### Anterograde manganese transport was impaired along the visual pathway of D2 mice with increasing IOP

**Figure 6:**
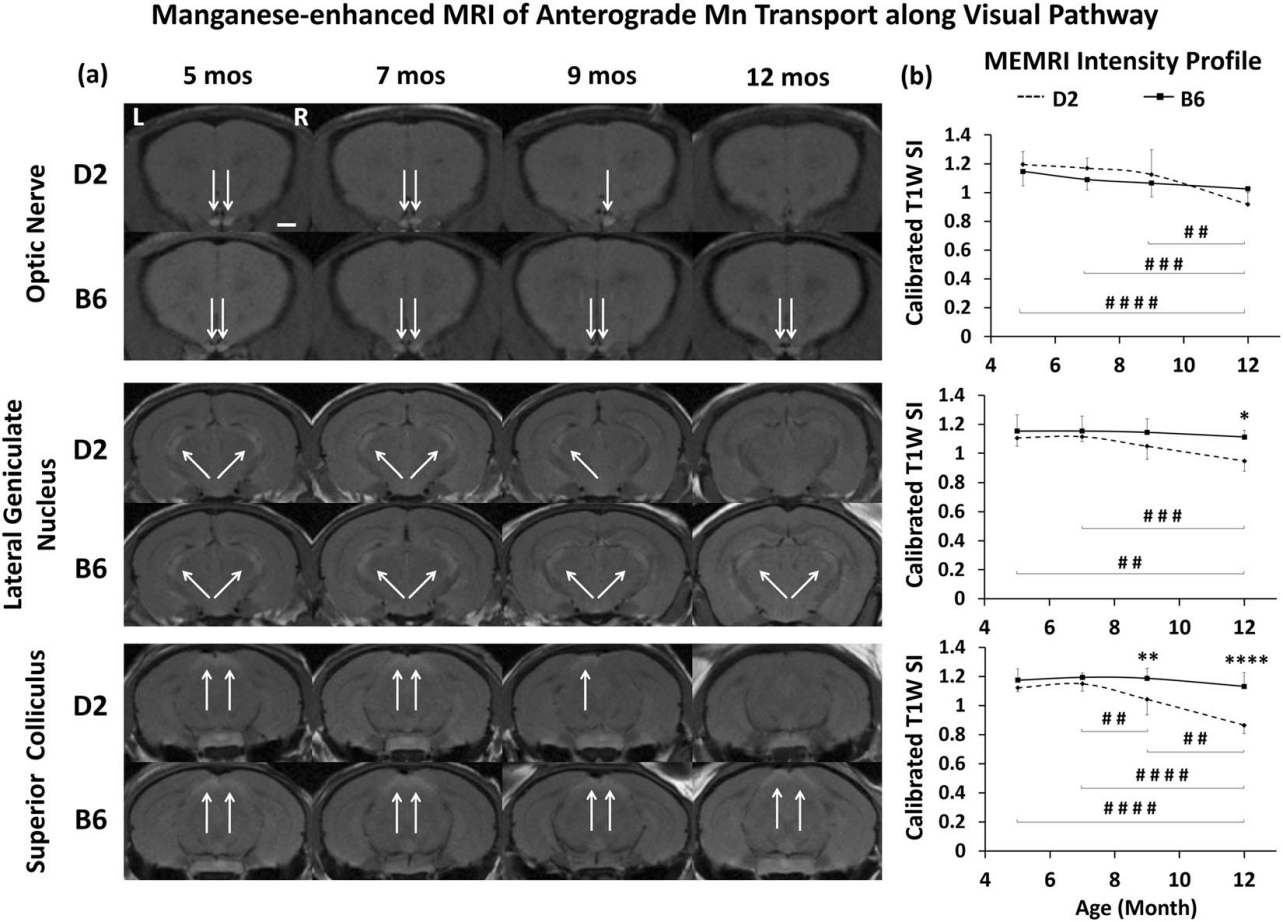
Manganese (Mn)-enhanced MRI of anterograde Mn transport along the visual pathway of D2 and B6 mice in Groups 1–4 across age. (**a**) Mn-enhanced MRI of the optic nerve (top 2 rows), lateral geniculate nucleus (middle 2 rows) and superior colliculus (bottom 2 rows) at 8 hours after intravitreal MnCl_2_ injection into both eyes at 5, 7, 9 and 12 mos. White arrows indicated Mn enhancements. Scale bar = 1 mm. (**b**) Spatiotemporal T1-weighted (T1W) signal intensity (SI) profiles in the optic nerve (top), lateral geniculate nucleus (middle) and superior colliculus (bottom) of the D2 and B6 mice across age. T1W SI was calibrated to a nearby saline phantom to account for potential system instability between Mn-enhanced MRI experiments. Post–hoc Tukey’s tests between ages in D2: ^#^p < 0.05, ^##^p < 0.01, ^###^p < 0.001, ^####^p < 0.0001; between D2 and B6: *p < 0.05, **p < 0.01, ****p < 0.0001. No significant difference was observed between ages in the B6 mice. Error bars represent ± standard deviation.

In the Mn-enhanced MRI assessments (Fig. 6), upon intravitreal MnCl_2_ injection into both eyes at 5 and 7 mos, D2 and B6 mice showed similar extents of T1-weighted Mn signal enhancement along the visual pathway in the prechiasmatic optic nerve, lateral geniculate nucleus and superior colliculus. D2 mice showed reduced Mn enhancement at 9 mos and almost no enhancement at 12 mos, whereas Mn enhancement remained observable in the B6 mice at 9 and 12 mos. Quantitatively, significant reduction in Mn enhancement was initially detected in the superior colliculus of the D2 mice at 9 mos, which further progressed at 12 mos. The optic nerve and lateral geniculate nucleus also showed significant Mn enhancement reduction at 12 mos in the D2 mice. No significant difference in Mn enhancement was observed along the visual pathway of the B6 mice across age. No significant difference in T1-weighted signals was observed along the visual pathway of either D2 or B6 mice before intravitreal Mn injection across age (data not shown).

## Discussion

D2 mice serve as a commonly used model for age-related pigmentary glaucomatous degeneration^10,12,13^. Previous histological studies on D2 mice showed optic nerve degeneration following initial IOP transition at about 8–9 mos, with the majority of the optic nerves being severely damaged by 12 months of age^12^. Using tonometry, we confirmed the onset and evolution of IOP elevation in D2 mice at ages consistent with those reported in the literature^12^. In addition, *in vivo* multi-parametric MRI assessments revealed the extent of early eye and brain changes in the D2 mice, some of which progressed along with increasing IOP elevation. MRI differentiation of these characteristic changes in the experimental glaucoma model may provide a non-invasive setting to determine early glaucoma mechanisms more specifically, to monitor different pathophysiological events in the visual system, and to evaluate glaucoma treatment strategies increasingly targeting both the eye and the brain in a more comprehensive way.

Recent clinical studies indicate that increasing axial length of the eye may be a risk factor for glaucoma^23^. Our serial ocular anatomy MRI measurements corroborated the sensitivity of axial length as a marker that paralleled IOP elevation in the D2 mice across age. Consistent with a recent *in vivo* optical coherence tomography study^24^, our ocular T2-weighted MRI indicated a greater elongation of axial length in the D2 mice than B6 mice throughout the experimental period. In addition, we found that both the anterior chamber and vitreous body deepened and drove the initial axial length increase during initial IOP elevation at about 9 mos. Early stage axial length increase had been considered as a potential compensatory reaction of the ocular wall that may trigger retinal ganglion cell loss and optic nerve head injury^25–27^, and our results indicate that both the anterior chamber and vitreous body may be involved in such initial response. Upon further increase in IOP elevation at 12 mos, the anterior chamber, but not the vitreous body, changed along with the residual increase in axial length. Given that successful glaucoma surgery is often accompanied with axial length decrease^28^, the combined measurements of IOP and individual ocular dimensions may provide an early indicator of glaucoma risk, leading to improved early diagnosis and treatment, and providing a consistent way to monitor therapy.

Glaucoma is characterized by retinal ganglion cell pathology and a progressive loss of vision. Initial evidence also suggests that optic nerve axon dysfunction and degeneration may precede neuronal loss in glaucoma^6,29,30^. DTI has been used to quantitatively depict the organization and connectivity of white matter microstructures in the brain, thus offering means to evaluate the spatiotemporal profile of visual pathway integrity in chronic glaucoma^15,31^. Despite a growing body of evidence indicating compromised optic nerve and optic radiation integrity in clinical DTI studies of glaucoma relying on fractional anisotropy as a non-invasive marker^32–36^, the exact pathophysiological events that drive the observed fractional anisotropy changes remain controversial. Comparing along the visual pathway, there were larger directional diffusivity changes in the optic nerve than the optic tract in D2 mice, suggesting that more severe structural alterations took place in the anterior visual pathway than in the posterior visual pathway over the experimental period. On the other hand, recent rodent DTI studies on transient retinal ischemia and excitotoxic retinal injury attributed the progressive fractional anisotropy decrease in the visual pathway to early axial diffusivity decrease and delayed radial diffusivity increase^14,15^. Our DTI study on the D2 mice showed that both fractional anisotropy and radial diffusivity in the optic nerve and optic tract significantly changed with IOP elevation at 9 and 12 mos, whereas significant axial diffusivity decrease was only observed at the end experimental time point at 12 mos. This pattern is consistent with our preliminary DTI analysis in a chronic glaucoma rat model, where fractional anisotropy and radial diffusivity but not axial diffusivity were significantly altered in the optic nerve early after experimental induction of chronic IOP elevation via laser-induced photocoagulation^37^. These findings suggest that chronic IOP elevation may lead to detectable spatiotemporal DTI characteristics in the extra-ocular structures of the visual system that are distinct from those found with direct, acute retinal injuries.

Early axial diffusivity and delayed radial diffusivity changes in the optic nerve have been demonstrated to be sensitive to early axonal degeneration and delayed demyelination, respectively, after transient retinal ischemia^14^. However, the different patterns of diffusional changes along the visual pathway in chronic experimental glaucoma indicate a complex interplay of neurodegenerative processes beyond axonal and myelin injuries. For example, in the presence of neurofilament and myelin sheath damage in the D2 mice after IOP elevation, recent immunohistochemical studies also showed the presence of nucleated cell infiltration, reactive astrogliosis, and inflammatory responses from microglial or macrophage upregulation in the optic nerve across age^38,39^. Inflammation and gliosis may alter the mean diffusivity as well as both axial and radial diffusivities^40–42^, which may in turn compensate any concurring axial diffusivity decrease from axonal injury. To improve the specificity of detecting the pathophysiological mechanisms underlying the diffusional changes in glaucoma, future studies may utilize higher-order diffusion models^41–43^ to help differentiate the inflammatory and glial responses from axonal and myelin damage by separating the isotropic diffusion changes from anisotropic diffusion changes. Diffusion MRI findings may be combined with other MRI modalities such as magnetization transfer MRI, which is sensitive to demyelination and inflammation^44,45^ (see Supplementary Fig. S1). Higher spatial resolution imaging may be used to determine any regional differences along the visual pathway in glaucoma^46^. Future studies may also examine the potential involvement of trans-neuronal degeneration in the visual cortex by diffusion kurtosis MRI, since this extended imaging technique can represent water diffusion properties more precisely than DTI alone and is potentially more sensitive to microstructural complexities, particularly in the gray matter^47^.

Apart from probing structural integrity by diffusion-based MRI measures, Mn-enhanced MRI allows *in vivo* evaluation of the physiological anterograde Mn transport between the retina and the brain’s visual pathway without depth limitation^18,48–51^. The observed anterograde Mn transport reduction during increasing IOP elevation in the D2 mice generally concurred with the increasing severity of neurofilament loss, tauopathy and inflammation in the glaucomatous visual pathway across age^38,39,52^, all of which may affect active anterograde axonal transport^53,54^. Tau accumulation in the optic nerve of D2 mice also signifies a common pathological hallmark for neurodegenerative diseases^53^. Our previous Mn-enhanced MRI and DTI experiments indicated that anterograde Mn transport is relatively more sensitive to axial diffusivity changes than radial diffusivity changes in the injured visual pathway^15^. Interestingly, both Mn enhancement and axial diffusivity in the D2 optic nerve changed significantly at 12 mos only, whereas reduced Mn enhancement in the superior colliculus of the D2 mice was detected earlier than in the optic nerve at 9 mos. These findings support a recent hypothesis that distal transport deficits occur early in the D2 mice^30^. While optic nerve fibers in rodents project mainly to the superior colliculus^55,56^, significantly reduced Mn enhancement was also detected in the lateral geniculate nucleus of D2 mice at 12 mos. Mn-enhanced MRI may offer an *in vivo* imaging model system to monitor therapeutic effects targeting intraretinal uptake and transport deficits in both the anterior and posterior visual pathways in future longitudinal studies^16,46,57–61^. No difference in anterograde Mn transport was observed between young and older B6 mice, which is consistent with a recent axonal transport study using cholera toxin beta injection in the same strain^62^.

To understand the effects of eye and brain changes on visual performance, we measured the visuomotor behavior of the D2 and B6 mice, and found that the B6 mice performed optokinetically better than the D2 mice at all ages. In addition, the behavioral experiments in this study showed a decline in visual acuity as early as at 7 mos, in contrast with other experiments in this study, which did not demonstrate significant changes until at least 9 mos. These results may reflect other early stage changes in the visual system, to which the presently used modalities were not sensitive^63,64^. For example, DTI showed that D2 mice exhibited a trend of lower fractional anisotropy and higher radial diffusivity in the brain’s visual pathway than the B6 mice even before IOP elevation (Fig. 5), suggestive of intrinsic brain differences between the two strains such as the involvement of early microglial activation and redistribution in the D2 mice^63,65^. It is essential to exert caution when trying to directly infer the visuomotor deterioration of glaucoma, as the D2 mice have been suggested to exhibit a limited intrinsic optomotor head-turning reflex^66–68^. From our experience, the performance of the D2 mice varied, and some D2 mice required a longer period of habituation in order to achieve more robust tracking responses. On the other hand, the young B6 mice had slightly lower IOP than the young D2 mice, whereas the older B6 mice had slightly higher IOP than the young B6 mice in consistency with recent findings^22^. Previous studies had attributed the different susceptibility of IOP-induced glaucomatous damages to the baseline properties in the eye across age and animal strains^22,69^. Future studies may utilize more comprehensive functional and behavioral assessments^68^ to determine how baseline differences in the eye and the brain may affect glaucomatous changes and visual performances in experimental glaucoma across age, gender, species and strains.

There are several limitations to the present study, the first being that wild type B6 mice are not genetically matched with D2 mice. Since both mouse strains are commonly used for modeling experimental glaucoma, yet the effect of age on the visual pathway of either D2 or B6 mice has not been well examined^22,25^, we documented in parallel the *in vivo* characteristics of the young and older D2 and B6 mice before any surgical or therapeutic interventions as the first steps to provide a foundation of the age-related changes for further experiments. At the same time, the main focus of our current study was on the age- and IOP-related changes in the same animals, or animals from the same species. While both direct and indirect comparisons of D2 and B6 mice have been used in this and other MRI- and non-MRI based studies of visual pathway changes^17,24,30,70–78^, future studies should also consider comparing D2 mice with the age- and genetically-matched DBA/2J-Gpnmb+/SjJ mice, both male and female. On the other hand. one important limitation of the Mn-enhanced MRI techniques used in this study is the range of potential determinants of Mn signal changes, including axonal transport, gray matter uptake, and extracellular diffusion^79^. While retinal apoptosis^80,81^, retinal ganglion cell loss^82^, and altered aqueous humor dynamics^19,60^ could also affect intraretinal Mn uptake^16^, progressive deficits in active anterograde transport have been shown histologically at 8 to 12 mos in the D2 mice by intravitreal cholera toxin beta injection^30,57,83^. In future studies, longitudinal scans over hours will be taken after intravitreal Mn injection to determine the rate of Mn transport along the visual pathway. Finally, the Mn enhancement in Fig. 6 showed lateralized deficit at 9 mos in D2 mice which progressed to bilateral deficit at 12 mos, whereas visual inspection of the DTI images in Group 1 also indicated apparent asymmetry of DTI metrics in the bilateral optic nerves in 0, 1, 3 and 5 mice out of 13 mice at 5, 7, 9 and 12 mos, respectively. Nevertheless, Mn-enhanced MRI and DTI experiments were performed in different sets of animals, which limited direct one-to-one comparisons that could be made among these modalities. In order to determine more specific biological relevance to the observed MRI changes, subsequent studies may involve quantitative analyses and correlations with multi-parametric MRI over the visual system with larger samples and more advanced modeling on a single animal group, allowing for more accurate comparison of the results. Other possible avenues of investigation could combine the current MRI modality with retinal and scleral imaging^15–17,84^ as well as electrophysiological and behavioral assessments^68^ for more in-depth monitoring of the eye-brain-behavior relationships and the progression of glaucoma in the visual system.

The current experiments have implications that are relevant to one of the most important goals in studying glaucoma: the testing and refinement of therapeutic options. Of particular value may be the experimental paradigm in this study, which has allowed for both longitudinal observation and examination of mice at specified endpoints. While current medical options are essentially limited to control of IOP, the present experiments have elucidated degenerative processes in both the eye and the brain that could prove targetable for early intervention in the management of glaucomatous change. Research within this field may pave the way to future therapeutic possibilities. The wide variety of experimental methods in this study, ranging from basic tonometry and visuomotor behavioral tasks to multi-parametric MRI, provide many avenues to explore the efficacy of neuroprotective treatment^58,61,64,85–88^ within a well-controlled animal model.

In conclusion, this study documents a non-invasive model system for dynamic monitoring of the onset and progression of eye, brain and behavioral changes in the D2 mouse model of inherited glaucoma and in wild type B6 mice across age. The occurrence of defects in the visual system of D2 mice generally coincides with a significant elevation of IOP, while continued high IOP might result in increasing severity of structural and physiological changes in the eye and brain’s visual pathway as the mice age. Our findings demonstrate a complex interplay between age and IOP as compared with the structure and function of the visual system in experimental glaucoma. The anterior and posterior visual pathways of the D2 mice exhibited differential susceptibility to glaucomatous neurodegeneration, which suggested the need to examine and target the visual system comprehensively in both the eye and the brain’s visual pathways for more effective prevention of glaucoma progression and vision loss.

## Methods

### Animal preparation

All experimental protocols were approved by the University of Pittsburgh’s Institutional Animal Care and Use Committee (Protocol number: 15086542) in accordance with the ARVO Statement for the Use of Animals in Ophthalmic and Vision Research. Thirty-one D2 and 19 B6 female mice were obtained from the Jackson Laboratory (Bar Harbor, ME, USA) and maintained in an AAALAC-accredited animal facility with a 12 hr light/dark cycle with standard rodent chow available *ad libitum*. All animals were divided into 4 groups as outlined in Fig. 1. In brief, both D2 and B6 mice in Group 1 underwent longitudinal ocular T2-weighted MRI and brain diffusion tensor MRI (DTI) at 5, 7, 9 and 12 mos, and D2 and B6 mice in Groups 1, 2, 3 and 4 underwent optokinetic assessments followed by manganese (Mn)-enhanced MRI or histology at 12, 5, 7 and 9 mos, respectively. IOP of all animals was measured before each MRI experiment. In Group 1, additional IOP measurements were performed in between 7, 9 and 12 mos for more precise longitudinal monitoring of the progression of IOP elevation.

### Intraocular pressure measurements

IOP levels in both eyes of all mice were measured using the TonoLab rebound tonometer (Colonial Medical Supply, Fransonia, NH, USA) under isoflurane gas anesthesia. At least 18 valid IOP values from each eye were obtained within 5 min after induction of general anesthesia and the values were averaged^19^.

### Visuomotor behavioral assessments

An OptoMotry virtual-reality system (CerebralMechanics, Lethbride, Alberta, Canada)^89,90^ was used to assess the visuomotor behavior in awake, freely moving animals by quantifying the visual acuity of each eye. All animals were first habituated in the optokinetic testing device before experiments. The optokinetic response was measured starting with a low spatial frequency sine wave grating at 0.03 cycle/degree with a constant rotation speed of 0.12 degree/s and 100% contrast. Clockwise and anti-clockwise rotations were presented in random orders to examine the visuomotor function of the left and right eyes, respectively^90^. The spatial frequency of the grating was incrementally increased until the animals failed to respond. The visual acuity was identified as the highest spatial frequency that the mice could track.

### MRI protocols

All MRI experiments were performed under isoflurane anesthesia using a 9.4-Tesla/31-cm Varian/Agilent horizontal bore scanner (Santa Clara, CA, USA) with a 32 mm transmit-receive volume coil. The mice were anesthetized by inhaling a mixture of air and isoflurane (3% for induction and 1.5% for maintenance). Artificial tears were applied to keep both eyes moist during scans. Respiration and body temperature were monitored, and core temperature was maintained with a circulating warm-water blanket.

To ensure reproducible slice orientation and positioning, scout images were first acquired in coronal, transverse and sagittal planes with a spin-echo pulse sequence. For anatomical ocular MRI, T2-weighted imaging was acquired using a fast spin-echo sequence with repetition time/echo time of 2000/42.4 ms, echo train length of 8, field of view: 14 × 14 mm^2^, acquisition matrix: 192 × 192, slice thickness of 0.5 mm and number of averages at 20. The slices were oriented to bisect the center of both the eyes and the optic nerves. DTI was acquired using a fast spin-echo sequence, with 12 diffusion gradient directions at diffusion weighting factor, *b*, of 1.0 ms/μm^2^ and 2 non-diffusion-weighted images at *b* = 0 ms/μm^2^ (*b*_0_). Other imaging parameters included: repetition time/echo time: 2300/27.8 ms, echo train length: 8, duration of diffusion gradient pulses (δ)/time between diffusion gradient pulses (Δ) of 5/17 ms, field of view: 2.0 × 2.0 cm^2^, acquisition matrix 192 × 192 (zero-filled to 256 × 256), slice thickness of 0.5 mm and number of averages at 4. The slices were oriented orthogonal to the prechiasmatic optic nerves.

At the experimental end point, a subset of the D2 and B6 mice from each group received intravitreal injection of 0.5 μL of 100 mM manganese chloride (MnCl_2_) solution into both eyes. Mn-enhanced MRI was performed before and 8 hrs after MnCl_2_ injection using a T1-weighted fast spin-echo sequence, with the same geometric parameters as DTI, repetition time/echo time of 1060/9.35 ms and echo train length of 4. A saline syringe phantom was placed next to the mouse head for signal normalization to account for potential system instability between imaging sessions.

### Data analysis

For ocular anatomical T2-weighted MRI, ocular dimensions including anterior chamber depth, vitreous body depth and axial length were measured in each eye using ImageJ v1.47 (Wayne Rasband, NIH, Bethesda, MD, USA).

For DTI, co-registration between non-diffusion-weighted b_0_ images and diffusion-weighted images was performed using SPM8 (Wellcome Department of Imaging Neuroscience, University College, London, UK). Using DTIStudio v3.02 (Johns Hopkins University, Baltimore, MD, USA), 3 × 3 diffusion tensors were fitted on a pixel-by-pixel basis from the non-diffusion-weighted b_0_ images and the diffusion-weighted images. The eigenvectors and eigenvalues of the diffusion tensors were derived to compute the DTI parametric maps including fractional anisotropy (FA) directionality color map, FA value map, axial diffusivity (λ_//_) map and radial diffusivity (λ_⊥_) map. Regions of interest were drawn manually using ImageJ at the center of the prechiasmatic optic nerves at Bregma 1.0 mm (4 voxels), the optic tracts at Bregma −1.7 mm (5–6 voxels), and the anterior commissure at Bregma 1.0 mm (4 voxels) based on the mouse brain atlas^91^ and the FA directionality map, FA value map, and λ_//_ and λ_⊥_ maps at each age.

For Mn-enhanced MRI, regions of interest were drawn manually using ImageJ on the prechiasmatic optic nerves at Bregma 1.7 mm, the lateral geniculate nuclei (LGN) at Bregma −2.3 mm and the superior colliculi (SC) at Bregma −3.4 mm based on the T1-weighted images and the mouse brain atlas. The T1-weighted signal intensities pre- and post-Mn injection were extracted and normalized to the signal intensity of the nearby saline syringe phantom.

IOP, ocular dimensions, DTI parametric values, calibrated Mn-enhanced MRI signal intensities and visual acuity were compared across age and between D2 and B6 mice using analyses of variance (ANOVA) followed by post-hoc Tukey’s multiple comparisons correction tests via GraphPad Prism v5.00 (GraphPad Software Inc., La Jolla, CA, USA). Quantitative data were presented as mean ± standard deviation unless otherwise specified. Results were considered statistically significant when p < 0.05.

## Electronic supplementary material

Supplementary Information
