# Supplementary material for: Age-related Changes in Eye, Brain and Visuomotor Behavior in the DBA/2J Mouse Model of Chronic Glaucoma
Source: Sci Rep. 2018 Mar 15;8:4643. doi: 10.1038/s41598-018-22850-4 (PMC5854610; doi:10.1038/s41598-018-22850-4)
Supplement: Supplementary file 1 — Supplementary Information [file 41598_2018_22850_MOESM1_ESM.pdf]

**SUPPLEMENTARY INFORMATION**

**Title:** Age-related Changes in Eye, Brain and Visuomotor Behavior in the DBA/2J Mouse Model of Chronic Glaucoma

**Author list:** Xiao-Ling Yang, Yolandi van der Merwe, Jeffrey Sims, Carlos Parra, Leon C. Ho, Joel S. Schuman, Gadi Wollstein, Kira L. Lathrop, and Kevin C. Chan*

*Corresponding author

In addition to longitudinal examinations, pilot magnetization transfer MRI (MTI) was performed to animals in Group 1 at 12 months old (mos), in order to correlate with diffusion tensor imaging (DTI)-derived parameters in the prechiasmatic optic nerve. The imaging parameters included: 9.5 μT saturation pulses at +6000 Hz off resonance and 150 ms pulse length, repetition time/echo time = 1500/37.34 ms, and number of repetitions= 2. Both MTI and DTI shared the same slice geometry, with in-plane resolution= 104×104 $\mu m^2$ and slice thickness= 1 mm. Results showed that the optic nerve of D2 mice had significantly reduced magnetization transfer ratio (MTR) compared to the B6 mice (0.527±0.128 vs 0.639±0.087) (Unpaired t-test, $p<0.05$), whereas significant correlations were found between MTR and DTI-derived parameters (Supplementary Fig. S1). These results suggest that both DTI and MTI are sensitive to compromised visual pathway integrity in experimental glaucoma. MTI has been suggested to be sensitive to demyelination and inflammation. Future studies may include more detailed histological quantitation and correlations, to determine if combined DTI and MTI comparisons can improve the specificity of detecting demyelination and inflammation in glaucoma.

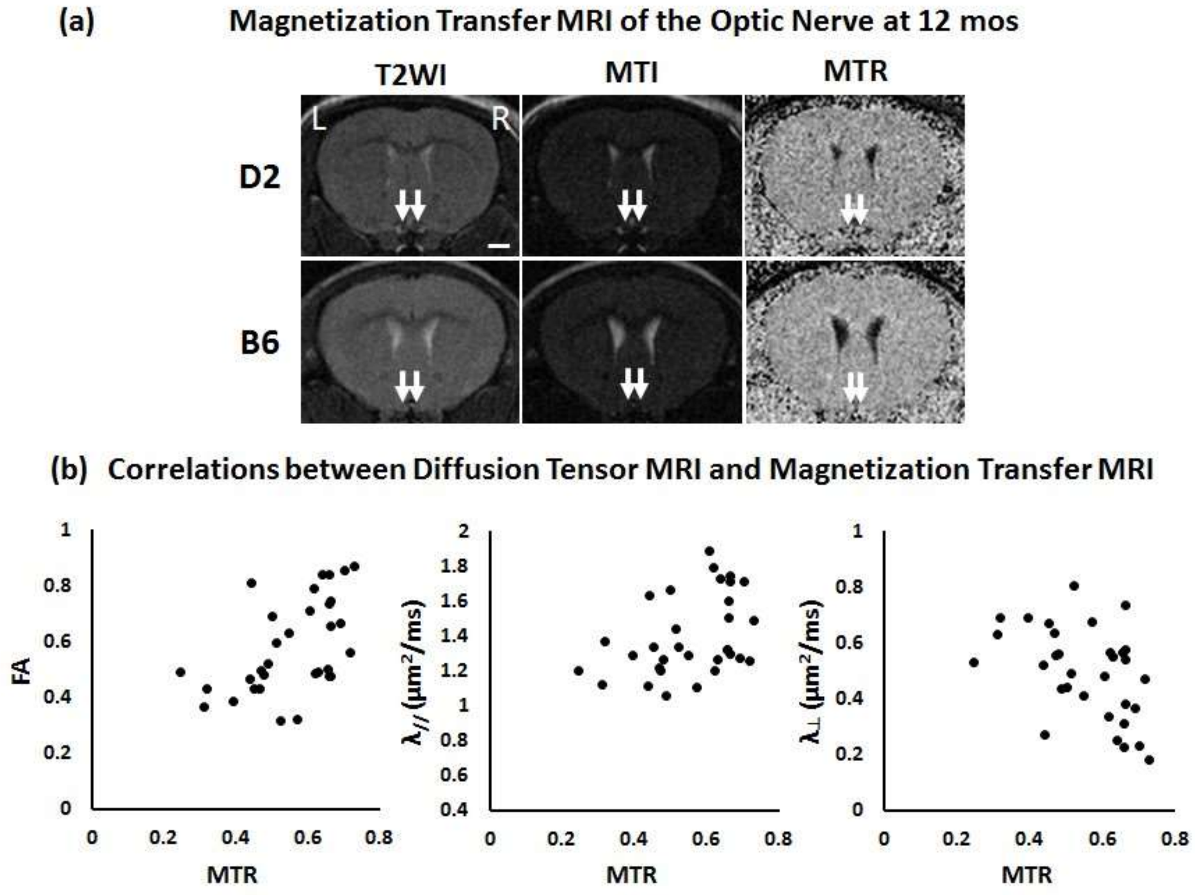


**Supplementary Figure S1: Magnetization transfer MRI at the level of the prechiasmatic optic nerve. (a)** T2-weighted MRI (T2WI), magnetization transfer imaging (MTI) and magnetization transfer ratio (MTR) mapping of both D2 and B6 mice at 12 months old (mos). Arrows indicate the prechiasmatic optic nerves. Scale bar = 1 mm. **(b)** Relationships between diffusion tensor imaging (DTI) parameters and MTR in the prechiasmatic optic nerve of Group 1 animals at 12 mos. Positive correlations were observed between MTR and fractional anisotropy (FA) ($r = 0.522$; $p<0.01$), and between MTR and axial diffusivity ($\lambda_{//}$) ($r = 0.403$; $p<0.05$), whereas a negative correlation was observed between MTR and radial diffusivity ($\lambda_{\perp}$) ($r = -0.449$; $p<0.01$).
